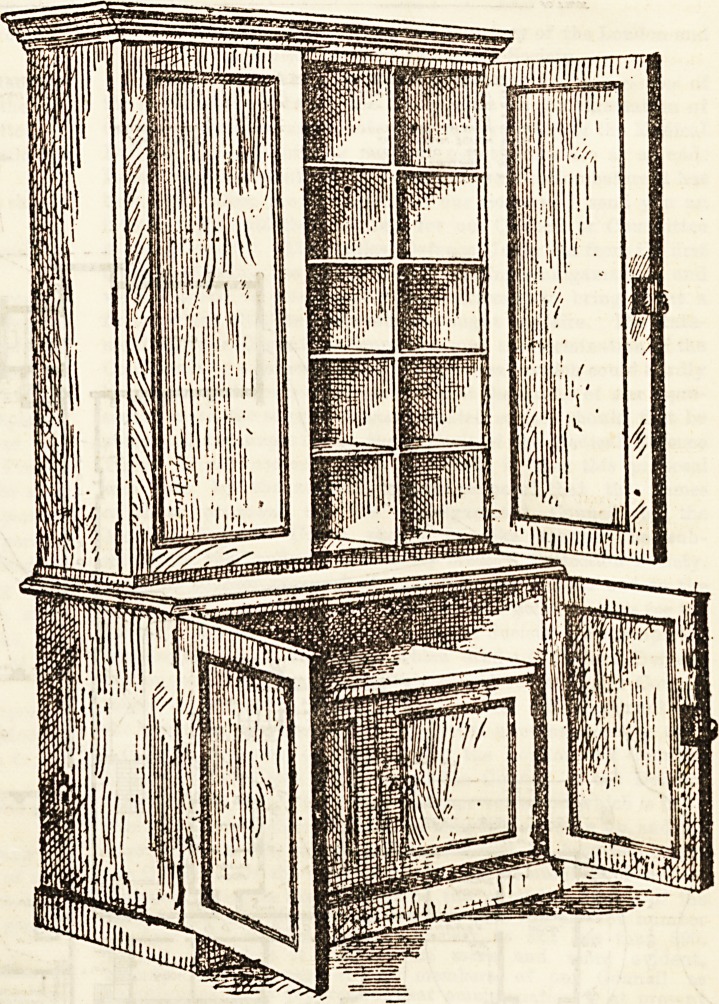# Ward Cupboards.—I. Medicine Cupboards

**Published:** 1893-12-02

**Authors:** 


### PRACTICAL DEPARTMENTS.

### WARD CUPBOARDS.—I. Medicine Cupboards.

The sister or head nurse of a hospital ward, if she be well up in her work, and methodical and orderly in her ways, takes much pride in her cupboards with their numerous stores of linen, bandages, and other appliances, medical and surgical. Adequate space for the due arrangement of those drugs which are kept in the ward, and for the safe bestowal of all poisons, is a necessary feature in the cupboard equipment. Where poisons are kept in the same receptacle with more innocent remedies, a separate division, which may be securely locked, is indispensable, the safe custody of all dangerous drugs being a point of the first importance, and one which will never be neglected by a careful nurse.

The medicine cupboard, of which we give an illustration, is made by Messrs. Atkinson, Westminster Bridge Road, and is roomy and useful. The upper portion is intended for ordinary medicines, lotions, &c., whilst the lower is provided with double doors for the safe reception of poisons. The space left between these two parts will be found very useful. Smaller cupboards, fixed against the wall at a convenient height, will of course in some cases be more convenient than so commodious an arrangement as the one shown here, and these in every variety may be procured from the same firm.

With regard to the best method of dividing the shelves, as such cupboards are usually made to order, it is best for those who are to use them to see to this being done in the manner most suited to their special requirements, which will vary in different cases. It is not a good plan to cut up the space too much for reasons of cleanliness and economy of room. Messrs. Atkinson's specialities in the way of hospital furniture are always to be relied on for good workmanship, and will be invariably found to be well-fitted and finished. Our drawing is by kind permission of the maker.

## Figures and Tables

**Figure f1:**